# Optimization of Q.Clear reconstruction for dynamic ^18^F PET imaging

**DOI:** 10.1186/s40658-023-00584-1

**Published:** 2023-10-20

**Authors:** Elisabeth Kirkeby, Lars Tore Gyland Mikalsen, Mona-Elisabeth Rootwelt-Revheim, Kyrre Eeg Emblem, Trine Hjørnevik

**Affiliations:** 1 Department of Physics and Computational Radiology, Division of Radiology and Nuclear Medicine, Oslo University Hospital, Building 20, Gaustad Sykehus, Sognsvannveien 21, 0372 Oslo, Norway; 2 Institute of Clinical Medicine, University of Oslo, Oslo, Norway; 3 Department of Life Sciences and Health, Oslo Metropolitan University, Oslo, Norway; 4 The Intervention Centre, Oslo University Hospital, Oslo, Norway; 5 Department of Nuclear Medicine, Division of Radiology and Nuclear Medicine, Oslo University Hospital, Oslo, Norway

**Keywords:** Dynamic PET, Quantitation, Recovery coefficient, β-factor, Q.Clear

## Abstract

**Background:**

Q.Clear, a Bayesian penalized likelihood reconstruction algorithm, has shown high potential in improving quantitation accuracy in PET systems. The Q.Clear algorithm controls noise during the iterative reconstruction through a β penalization factor. This study aimed to determine the optimal β-factor for accurate quantitation of dynamic PET scans.

**Methods:**

A Flangeless Esser PET Phantom with eight hollow spheres (4–25 mm) was scanned on a GE Discovery MI PET/CT system. Data were reconstructed into five sets of variable acquisition times using Q.Clear with 18 different β-factors ranging from 100 to 3500. The recovery coefficient (RC), coefficient of variation (CV_RC_) and root-mean-square error (RMSE_RC_) were evaluated for the phantom data. Two male patients with recurrent glioblastoma were scanned on the same scanner using ^18^F-PSMA-1007. Using an irreversible two-tissue compartment model, the area under curve (AUC) and the net influx rate K_i_ were calculated to assess the impact of different β-factors on the pharmacokinetic analysis of clinical PET brain data.

**Results:**

In general, RC and CV_RC_ decreased with increasing β-factor in the phantom data. For small spheres (< 10 mm), and in particular for short acquisition times, low β-factors resulted in high variability and an overestimation of measured activity. Increasing the β-factor improves the variability, however at a cost of underestimating the measured activity. For the clinical data, AUC decreased and K_i_ increased with increased β-factor; a change in β-factor from 300 to 1000 resulted in a 25.5% increase in the K_i_.

**Conclusion:**

In a complex dynamic dataset with variable acquisition times, the optimal β-factor provides a balance between accuracy and precision. Based on our results, we suggest a β-factor of 300–500 for quantitation of small structures with dynamic PET imaging, while large structures may benefit from higher β-factors.

***Trial registration*:**

Clinicaltrials.gov, NCT03951142. Registered 5 October 2019, https://clinicaltrials.gov/ct2/show/NCT03951142. EudraCT no 2018-003229-27. Registered 26 February 2019, https://www.clinicaltrialsregister.eu/ctr-search/trial/2018-003229-27/NO.

## Background

Positron emission tomography (PET) uniquely facilitates quantitation of physiological and pathophysiological processes in vivo. Absolute quantitation of a functional process requires a dynamic PET acquisition, to track the radiotracer kinetics (uptake, retention, and clearance) in tissue over time, and a measurement of the tracer concentration in arterial blood. The latter traditionally requires arterial cannulation, an invasive procedure; however, the use of image-derived input function (IDIF) is an alternative [1].

Several factors influence the quantitative accuracy in a complex dynamic imaging setup. Data are continuously collected in listmode and reconstructed into time frames of variable durations. Time frames are typically short in the beginning of the scan (5–10 s) and increase during scanning. During this period, the radioactivity concentration of the tracer in tissue also varies over time. The consequence is a dynamic data set consisting of frames with a combination of high- and low-count statistics. Another key factor is the limited spatial resolution of PET. State-of-the-art clinical PET/CT scanners provide a spatial resolution of 4–6 mm [2–4]. The quantitative accuracy of small structures will be affected by limited spatial resolution and partial volume effects (PVE) must be taken into consideration when analysing clinical data. For example, in neuroimaging, an IDIF can, if applicable, be extracted from intracranial blood vessels. The cerebral part of the internal carotid artery (ICA), which is commonly used, has a mean diameter of 4.66 ± 0.78 mm for women and 5.11 ± 0.87 mm for men [5] and can be difficult to segment due to limited spatial resolution and subjected to measurement errors [6].

PET images are commonly reconstructed by the widely clinically implemented ordered subset expectation maximization (OSEM) method [7]. A key shortcoming of OSEM is excessive noise with increasing number of iterations and insufficient convergence associated with underestimation of radioactivity concentration at insufficient iterations, particularly in small structures. Q.Clear, a Bayesian penalized likelihood iterative reconstruction algorithm (GE Healthcare), was developed to overcome these issues by controlling noise during iterations in order to allow full convergence [7]. The block sequential regularized expectation maximization algorithm suppresses noise by penalizing strong relative differences between voxels while preserving edges and also incorporates a point spread function (PSF) model. The strength of the noise-penalization is controlled by the parameter β which is the only controlling reconstruction parameter that is available to the user. Studies have shown that the use of Q.Clear resulted in improved contrast recovery (CR) and reduced noise compared to OSEM in both phantom and patient studies [8, 9]. Te Riet and colleagues demonstrated that increasing the β-factor improves signal-to-noise ratio, but at the cost of small lesion detectability in whole-body ^18^F-FDG scans [10]. To date, most published studies have focused on how the choice of β-factor affects image noise and lesion detectability [8, 11], and not on the quantitative accuracy of PET data. In addition, many phantom studies are based on the National Electrical Manufacturers Association (NEMA) image quality phantom containing spheres ≥ 10 mm diameter [12]. With the recent developments of modern digital PET systems and advanced image reconstruction algorithms, smaller spheres are required to fully evaluate the performance of novel reconstruction methods. Furthermore, studies have until now mainly focused on whole-body static PET scans. To the best of our knowledge, only one published study has investigated the effect of the choice of β-factor on the analysis of dynamic clinical PET data [13]. Ribeiro et al. compared the outcome parameter of a brain kinetic modelling analysis using different β-factors and a standard OSEM protocol. However, this was done only in clinical data and for a ^11^C-labelled tracer.

Here, we investigate how the β-factor in the Q.Clear algorithm affects quantitation and variability of tracer uptake in a dynamic ^18^F PET data set. The phantom study was designed to mimic a clinical dynamic PET scan (i.e. structures of various sizes, combination of frames of variable durations and repeated scans). In addition, two clinical cases were included to illustrate the impact of β-factor on the pharmacokinetic analysis of clinical ^18^F PET brain data.

## Materials and methods

### Phantom study

#### Phantom

**Fig. 1:**
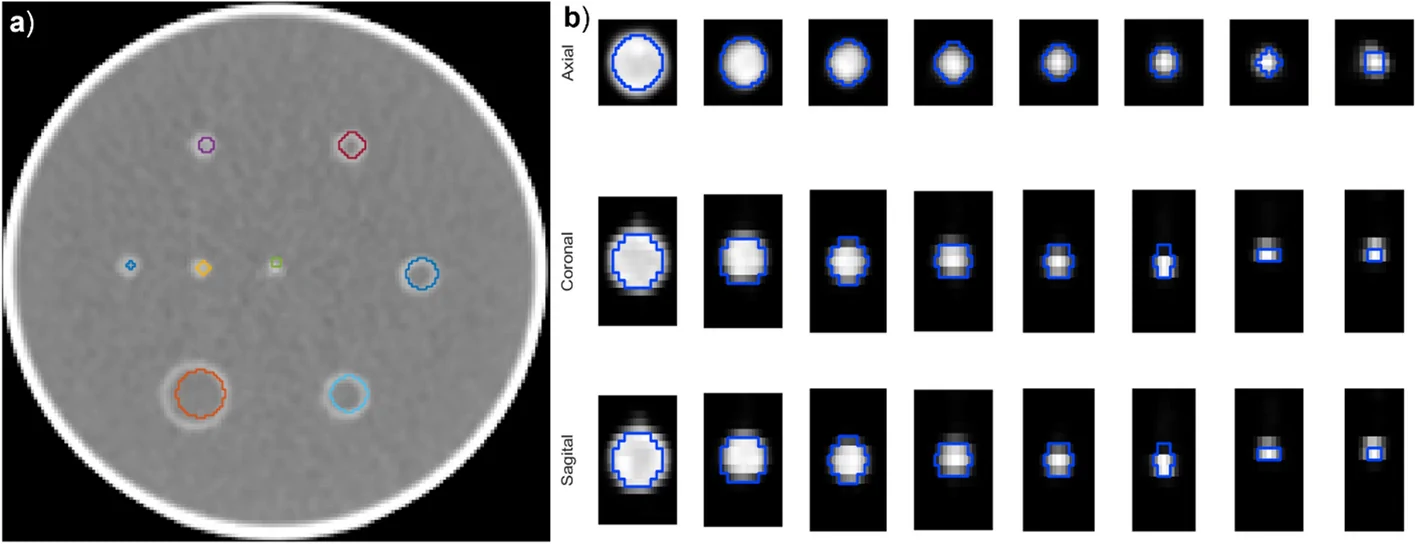
Example delineations of VOIs for each sphere shown on the CT (**a**) and PET image (**b**) in all three directions

A Flangeless Esser PET Phantom (model PET/FL/P) with hollow sphere sets ECT/HS/SET6 and ECT/MI-HS/SET4 (Data Spectrum Corporation, Hillsborough, NC, USA) was used in this study. The phantom has an inner diameter of 20 cm, and eight hollow spheres with inner diameter of 25, 15, 12, 10, 7.9, 6.2, 5.0 and 4.0 mm (volumes of 8.0, 2.0, 1.0, 0.5, 0.25, 0.125, 0.063 and 0.031 ml, respectively) were mounted in the phantom sorted according to diameter, with the six largest spheres in the circular positions 57.2 mm off centre, the second smallest spheres 28.6 mm off centre, and the smallest in the centre. No further inserts were used, see layout in Fig. 1. The spheres and the background compartment were filled with 17.2 kBq/ml and 1.6 kBq/ml ^18^F-FDG (ratio = 10.5), respectively.

#### Data acquisition and reconstructions

The phantom was placed on the table, centred using the positioning lasers and scanned on a Discovery MI PET/CT system (GE Healthcare, Milwaukee, WI, USA) with a 20-cm axial field of view (FOV) for 25 min. Data were collected in listmode and images were reconstructed to five 5-min frames (entire scan), five 2-min frames (from the first 10 min of the scan), five 1-min frames (from the first 5 min of the scan), five 30-s frames (from the first 2.5 min of the scan) and five 10-s frames (from the first 50 s of the scan). Images were reconstructed using Q.Clear (including time of flight (TOF), PSF modelling, a 256 × 256x71 matrix and 30 cm FOV) with 18 different β-factors ranging from 100 to 3500 and corrected for radioactive decay, dead time, attenuation, random coincidences and scattered radiation. In total, 450 reconstructed image series were reconstructed. A low-dose CT (120 kVp, 35 mA, revolution time 1, pitch 0.98, and slice thickness 0.625) was acquired for attenuation correction and structural information.

#### Data analysis

PET images were analysed using an in-house MATLAB-based program. First, a spherical volume of interest (VOI) matching the volume of each sphere was automatically placed around each sphere as identified on the CT data. Second, to correct for potential misalignment between the PET and CT data, the individual VOIs were allowed to be moved a maximum of one voxel in any direction to ensure inclusion of the maximum activity concentration in the PET data (Fig. 1). The latter procedure was applied individually for all 450 image series.

To quantify the measured activity concentration in the spheres, the average of the 10% hottest voxels in each VOI was calculated, from here on referred to as the peak value. The peak measurement was used to avoid the uncertainty of using the max SUV in each sphere, as the max value if affected by noise in the image. The recovery coefficient (RC), the coefficient of variation (CV_RC_) and the root-mean-square error (RMSE_RC_) were computed to assess the impact of different β-factors on the bias, variability and accuracy of quantitation. RC (Eq. 1) of each sphere states how accurately the known concentration is reproduced in a specific volume and is calculated as the ratio of the measured and known concentration [14]:

1 $$RC=\frac{\overline{{c }_{measured, peak}}}{{c}_{known}}$$

where $\overline{{c }_{measured, peak}}$ is the average of the measured activity concentrations in the five reconstructed repetitions, and c_known_ is the known activity concentration in the hot sphere. An RC of 1 suggests perfect reproduction of the activity concentration.

CV_RC_ (Eq. 2) gives an estimate of the variability for the calculated RC for the different spheres and β-factors. CV_RC_ is the ratio of the standard deviation of the RC ($${SD}_{RC})$$ for the five reconstructed repetitions and the mean of the RC defined as [15]

2 $${CV}_{RC}\left(\%\right)=100 x\frac{{SD}_{RC} }{RC}$$

RMSE_RC_ (Eq. 3) provides the difference between predicted and actual values and was calculated to investigate which β-factor would give the most accurate prediction for all sphere sizes and acquisition times. The RMSE_RC_ calculations include the RC value for each individual reconstructed repetition and all acquisition times. To investigate the potential impact due to sphere sizes, the RMSE_RC_ was calculated for all spheres combined, the four largest spheres (10–25 mm) and for the four smallest spheres (4.0–7.9 mm). An RMSE_RC_ of 0 would suggest a perfect result. A favourable β-factor range was defined based on the lowest RMSE_RC_ + 10%.

3 $${RMSE}_{RC}=\sqrt{\frac{{\sum }_{i=1}^{n}{(1-{c}_{measured, peak}/{c}_{known})}^{2}}{n}}$$

### Clinical evaluation

Data from two male patients with recurrent glioblastoma included in an ongoing clinical trial (NCT03951142) at Oslo University Hospital was retrospectively selected for this study. The clinical trial is approved by the National Research Ethics Committee and the Institutional Review Board (2017/1875), and all patients gave their written and informed consent.

#### Data acquisition and image reconstruction

^18^F-PSMA-1007 (2.5 MBq/kg) was injected intravenously and the patients were scanned dynamically for 30 min on a Discovery MI PET/CT system (GE Healthcare, Milwaukee, WI, USA). The PET data were reconstructed using Q.Clear (TOF, PSF modelling, a 256 × 256 matrix, 30 cm FOV) with a range of β-factors chosen from the results of the phantom study. Images were dynamically reconstructed into 32 frames of variable durations (18 × 10 s, 6 × 30 s, 4 × 1 min and 4 × 5 min) and corrected for radioactive decay, dead time, attenuation, random coincidences and scattered radiation. A low-dose CT (120 kVp, 35 mA, revolution time 1, pitch 0.98, and slice thickness 0.625) was used for attenuation and scatter correction.

#### Pharmacokinetic analysis

**Fig. 2:**
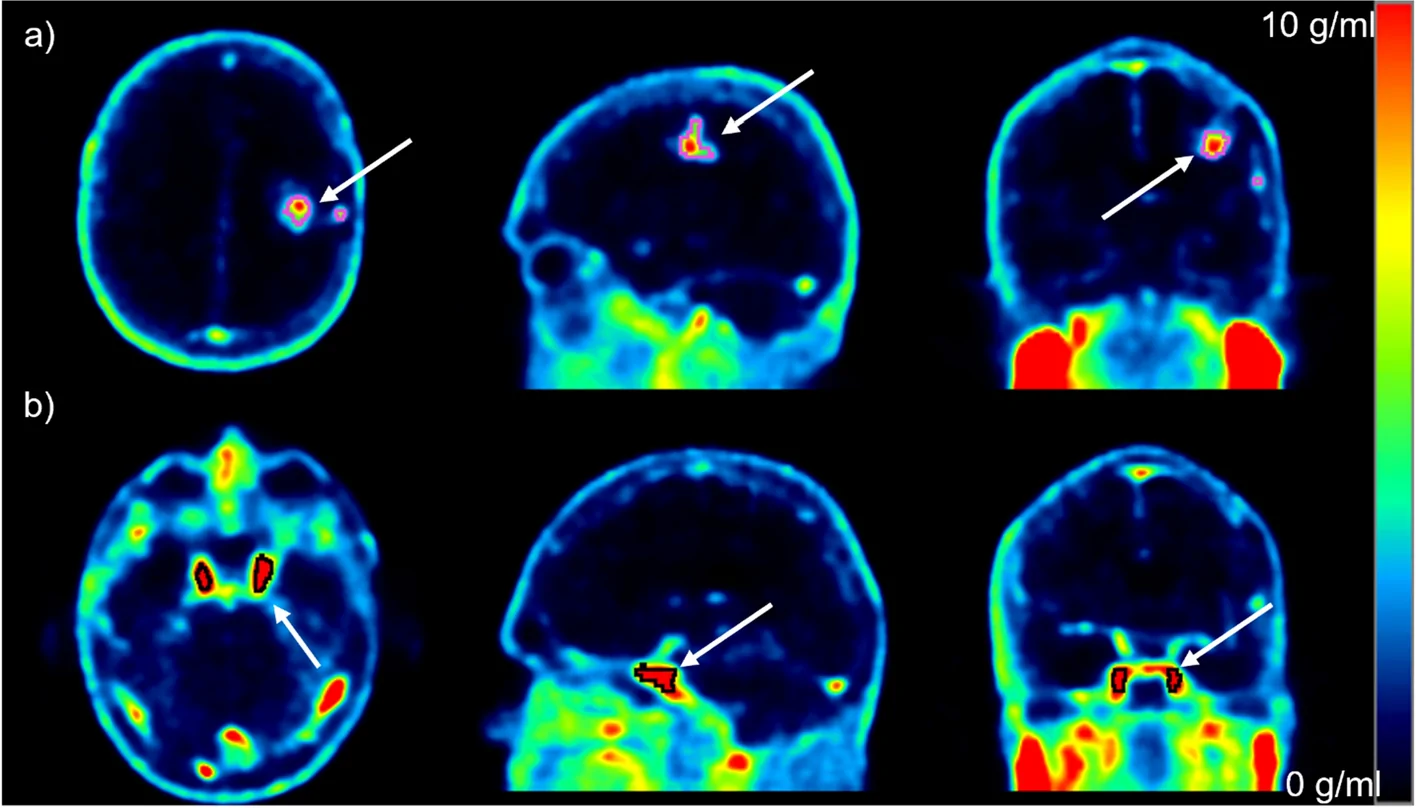
Standardized uptake value (SUV) image of a representative male adult patient with recurrent glioblastoma scanned with ^18^F-PSMA-1007. ^18^F-PSMA-1007 uptake and delineated VOIs are shown for tumour tissue (white arrow in **a**) and blood pool (white arrow in **b**)

Image analysis was performed using PMOD 4.0 (PMOD Technologies LLC, Zurich, Switzerland). First, to correct for patient motion during scanning, a rigid matching approach between frames was applied using the average uptake during the first two minutes of the scan as the reference image. For the pharmacokinetic modelling of the data, an IDIF was extracted as a proxy for an arterial input function. The IDIF was delineated by segmenting the bilateral ICA (threshold: 50% of max signal) on the blood pool image (first minute after injection of tracer). The uptake of ^18^F-PSMA-1007 in tumour tissue was extracted from an automatically delineated VOI using a threshold of 50% of max signal on a summed 20–30 min post-injection image. The blood pool and tumour tissue VOIs are shown in Fig. 2 for one of the included clinical cases.

Time activity curves (TACs) were extracted for the blood and tumour VOIs, and an irreversible two-tissue compartment model was used to calculate the net influx rate K_i_ of the tracer in the tumour tissue as defined by Eq. 6 [16]:

4 $${K}_{i}=\frac{{K}_{1} \times {k}_{3}}{{k}_{2}+{k}_{3}}$$

where K_1_ [ml/ccm/min] and k_2_ [1/min] are the uptake and clearance rate constants, and k_3_ [1/min] describes the trapping. The area under the curve (AUC) for the blood TAC and the tumour TAC was calculated to investigate the impact of the chosen β-factors on the kinetic analysis. In the absence of a gold standard, changes in AUC and K_i_ due to different β-factors were reported as the percentage differences between the lowest and highest β-factors used.

## Results

### Phantom study

**Fig. 3:**
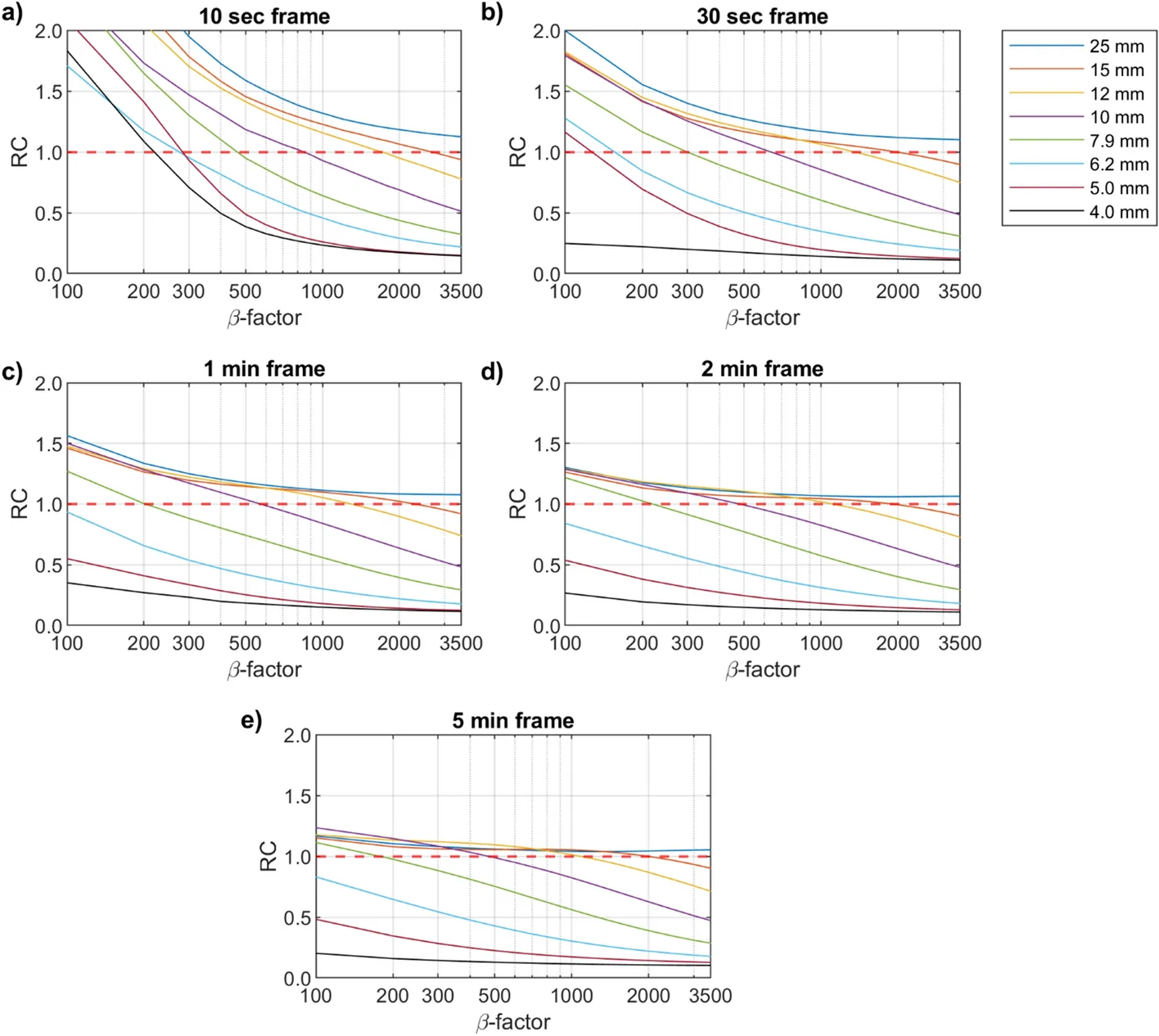
RC (mean of five measurements) versus β-factor for various sphere sizes and acquisition times. The red dotted line represents a ratio of measured and known concentration of 1

Figure 3 shows the RCs for all sphere sizes and acquisition times. In general, RC decreased with increasing β-factor. The use of lower β-factors tended to overestimate the measured activity concentration in data with low-count statistics (Fig. 3a, b). Increasing the β-factor (and hence the noise suppression) led to a smaller bias in the measurement for the larger spheres (≥ 10 mm), however at a cost of underestimating the measured activity concentration in the smaller spheres. The degree of over- and underestimation was highly dependent on acquisition time. For acquisition times ≥ 1 min, the measured activity concentrations in the smaller spheres (< 10 mm) were increasingly underestimated for β-factors ≥ 200 (Fig. 3c-d). As shown in Fig. 3e, even for data with high-count statistics, the bias of measurements was challenging in small spheres. Consequently, the use of a low β-factor was beneficial for reproducing the activity concentration in smaller structures. In contrast, a more mid-range β-factor provided the best results for larger spheres (≥ 10 mm).

**Fig. 4:**
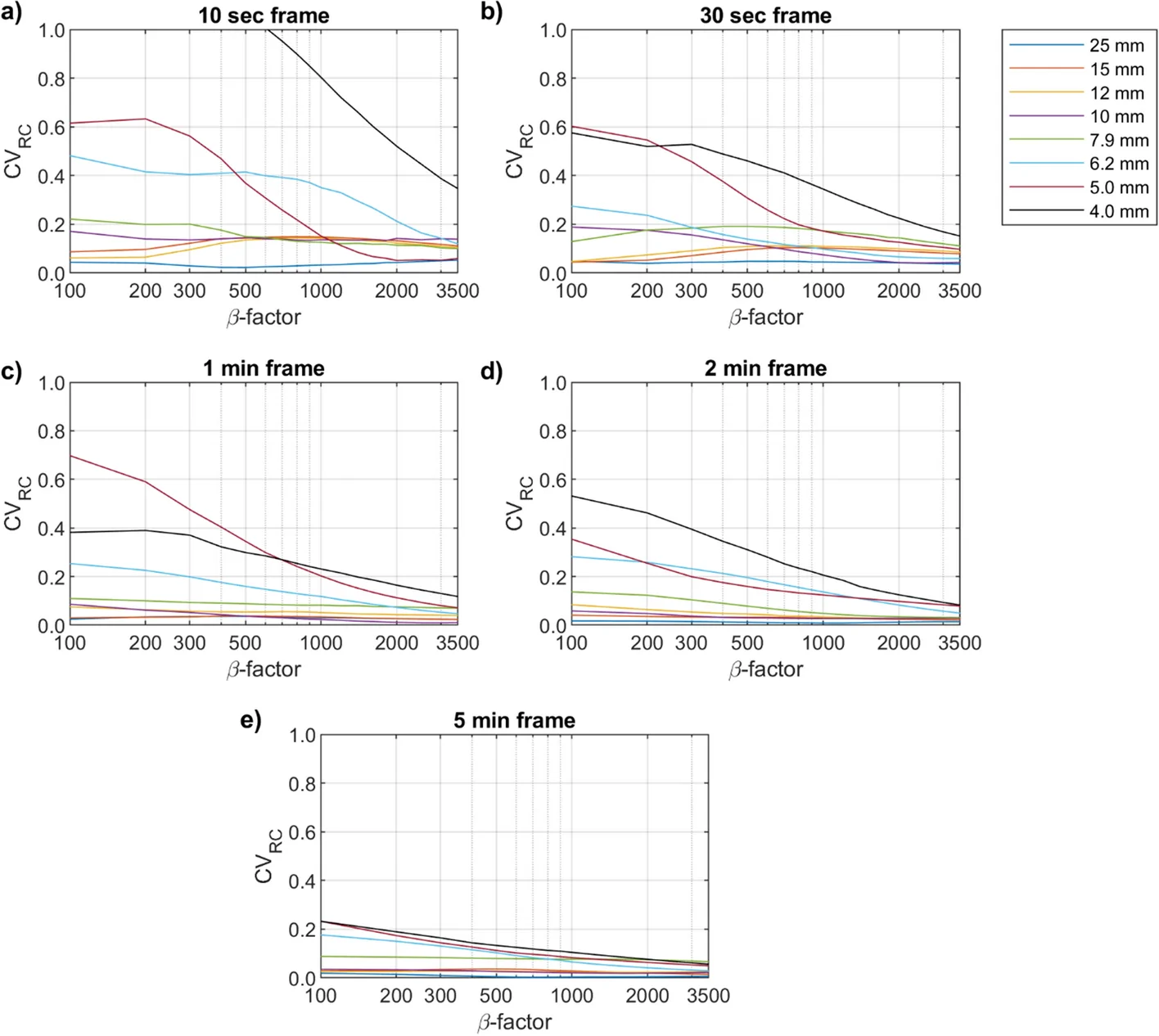
CV_RC_ versus β-factor for various sphere sizes and acquisition times

The variability measurements are shown in Fig. 4. In general, CV_RC_ decreased with increasing acquisition time and β-factors. A high variability was observed for the smallest spheres (< 10 mm), even in the high-count statistical data. For acquisition times up to 2 min, one or more of the small spheres gave a CV_RC_ above 40%, and the 5-min reconstructions provided values that mostly were below 20%. Increasing the β-factor improved the variability of these measurements, however, at the cost of bias (Fig. 3). For the larger spheres (≥ 10 mm), a variability of less than 20% was achievable at all acquisition times: less than 10% for the 1-min and 2-min acquisitions, and less than 4% for the 5-min acquisition, respectively.

**Fig. 5:**
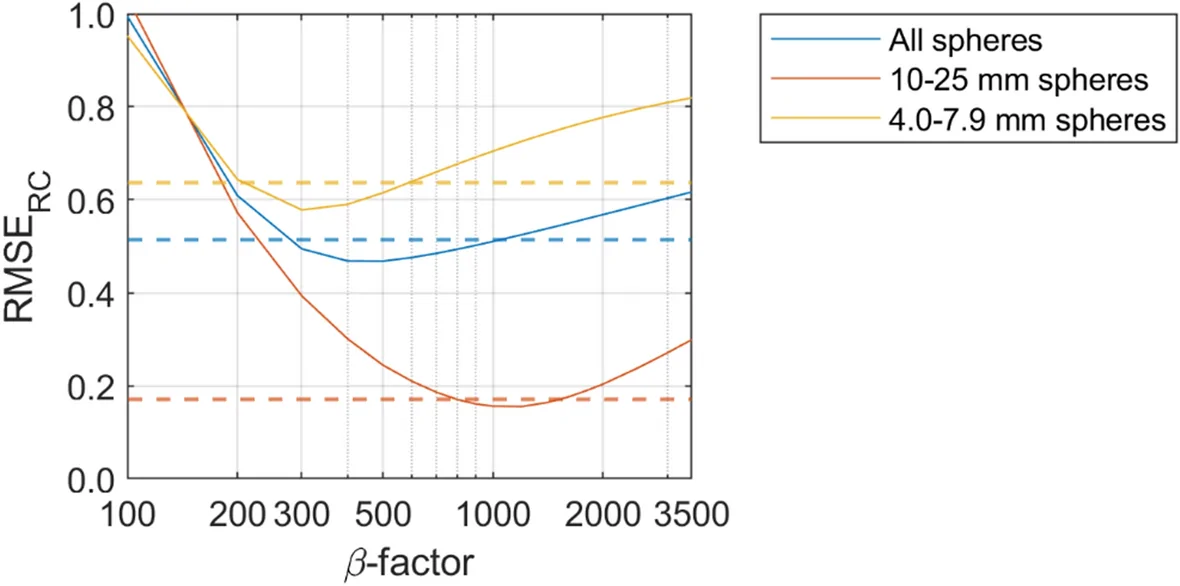
RMSE_RC_ versus β-factor for all spheres (blue), 10–25 mm spheres (red) and 4–7.9 mm spheres (yellow). The dotted lines represent a 10% change from the lowest RMSE_RC_ value

The RMSE_RC_ was calculated to assess the accuracy of measurements in a complex dynamic dataset with variable acquisition times. When combining all sphere sizes, a β-factor of 500 was found to give the most favourable results. If we allow for a 10% variation in RMSE_RC_, a favourable β-factor will be in the range of 300–1000 (Fig. 5). If optimizing the accuracy only for smaller structures (< 10 mm), a slightly lower β-factor of 300 gives the lowest RMSE_RC_ with a 10%-range of 200–600. Similarly to the RC results, the larger spheres (≥ 10 mm) would benefit from using a higher β-factor in the 800–1600 range, with 1200 providing the lowest RMSE_RC_.

### Clinical evaluation

**Fig. 6:**
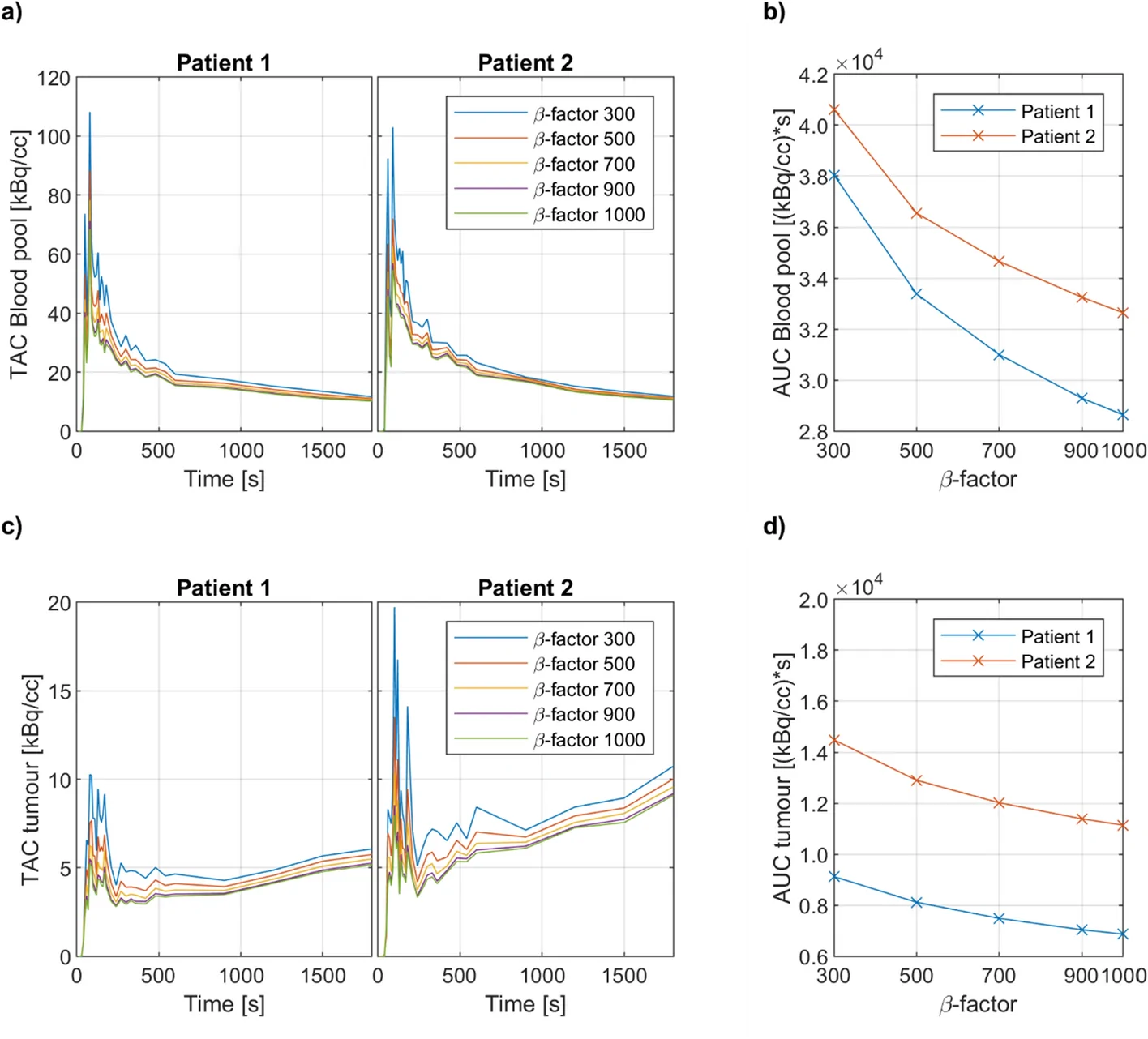
Time activity curves (TACs) for the blood pool and tumour VOIs are shown in panels a and c. AUC of blood and tumour TACs are shown in panels b and d for β-factors 300–1000 for both patients

The clinical PET data were reconstructed with β-factors 300–1000 based on the findings from the phantom study. The TACs for both blood pool and tumour VOIs are presented in Fig. 6 for both patients, together with the AUC. K1, k2, k3 and the estimated net influx rate (Ki) values from the kinetic analysis of the distribution of tracer for patient 1 and 2 are presented in Table 1. For both patients, the tumour volume increased with increasing β-factor. The tumour VOI had a volume of 3621–4695 mm^3^ and 1341–1883 mm^3^ for β-factors 300–1000 for patient 1 and 2, respectively. An increase in β-factor from 300 to 1000 resulted in a decrease in 24.7% and 19.6% for the blood TAC AUC, and 24.7% and 23.1% for the tumour TAC AUC for patient 1 and 2, respectively. In addition, the net influx rate of tracer increased with 30.8% and 20.2% for the two patients.

**Table 1:** K1 [ml/ccm/min], k2 [1/min], k3 [1/min] and Ki [ml/ccm/min] values from the kinetic analysis of the distribution of ^18^F-PSMA for patient 1 and 2

β- factor	Patient 1				Patient 2			
	K1	k2	k3	Ki	K1	k2	k3	Ki
300	0.348	4.002	0.082	0.007	0.550	4.974	0.114	0.012
500	0.369	4.847	0.108	0.008	0.758	7.961	0.142	0.013
700	0.312	4.774	0.134	0.009	0.643	7.994	0.178	0.014
900	0.290	4.809	0.155	0.009	0.547	8.000	0.220	0.015
1000	0.286	4.911	0.163	0.009	0.514	8.000	0.238	0.015

## Discussion

In this study, we assessed the impact of β-penalization factor on accurate quantitation of dynamic PET scans using a Flangeless Esser PET Phantom (PET/FL/P). We found that β-factor, acquisition time and structure size influence measurement bias, variability, and accuracy. We also explored how the β-factor affected the pharmacokinetic analysis of clinical dynamic PET brain scans, observing changes in input and outcome parameters. For precise quantitation of dynamic PET scans in small geometries, β-factors in the 300–500 range are recommended.

Dynamic PET datasets consist of time frames with varying durations and changing tracer concentrations, resulting in variable sets of images with both low- and high-count statistics, which influence the quantitative accuracy. To our knowledge, only one previous study has examined the β-factor’s impact on pharmacokinetic modelling in dynamic PET brain imaging [13]. The authors compared the use of Q.Clear with variable β-factors (100–1000 in increments of 100) was compared to OSEM-reconstructed data. A β-factor between 100 and 200 was recommended. However, the quantitative accuracy was not assessed using a phantom study, and the study was based on a ^11^C-labelled tracer which has a poorer intrinsic spatial resolution due to longer positron range of ^11^C compared to ^18^F-labelled tracers. Our data showed that RCs are strongly overestimated and highly variable at these low β-factors when short acquisition times are used, and we therefore advise that a β-factor of 200 and less should be avoided for ^18^F-labelled tracers.

RMSE_RC_ combines RC deviation (bias) and CV_RC_ (variability) to estimate overall accuracy. By averaging phantom results across sphere sizes and acquisition times, β-factors between 300 and 1000 provided similar accuracy, to within 10% of the minimal RMSE_RC_ (Fig. 5) with small and large spheres optimized at the ends of this range. For small structures (< 10 mm), a β-factor between 200 and 600 is favourable, but the RCs of small structures are very sensitive to β-factors within this range, in particular for short frame durations (Fig. 3). For example, for the 5-mm sphere with a 10-s acquisition time, changing the β-factor from 200 to 600 results in going from 40% overestimation to 60% underestimation of measured activity. Small β-factors enhance image detail, but supress less noise, leading to RC values exceeding 1 at short acquisition times, despite PVE. However, variability is high (Fig. 4). For instance, for the 10-s frame, the 5-mm sphere achieves nearly perfect RC at β-factor 300, but with a CV_RC_ above 50%. Increasing the acquisition time to 2 min reduces noise and results in a 62% and a 77% underestimation of the measured activity for β-factors 200 and 600, respectively. Importantly, this improves CV_RC_ to below 20% for β-factors starting at 300. Based on RC, a β-factor of 600 may be too high for small structures, but could be considered if low variability is of high importance (Figs. 4 and 5). Larger regions of interest can allow for the use of higher β-factors.

Teoh et al. [8] recommend a β-factor of 400 based on both phantom and clinical data. The phantom study included only one acquisition time and spheres ≥ 10 mm. The results are in accordance with the study by Tian et al*.* [17], where the authors also recommend a β-factor of 400. Caribé and colleagues [9] supplemented these studies by including five different acquisition times (1, 2.5, 5, 10 and 20 min) and showed that a β-factor of 300–400 is favourable for maximizing the CR for spheres ≥ 10 mm. These studies focus only on maximizing CR and lesion detectability, and not on the accuracy of the measurements which is the primary end point of quantitative imaging. With modern digital PET systems and advanced image reconstruction algorithms, the spatial resolution of PET has improved, and smaller structures should be included in phantom studies. For the PET/CT system tested in this study (Discovery MI PET/CT), the axial spatial resolution as measured following the NEMA NU2-2012 procedure is 3.9 mm [12]. We have therefore included spheres sizes in the range from 4 to 25 mm diameter. Our findings are comparable with the work by Miwa et al. [18], who assessed the impact of β-factors (range: 50–400) on the measurement bias for small structures ranging from 4 to 13 mm diameter. For a typical clinical acquisition time of 2 min, Miwa and colleagues reported that the RCs for β-factors between 50 and 100 were overestimated, whereas for β-factors between 300 and 400, the RCs were underestimated. They conclude that a β-factor of 200 is optimal for detecting sub-centimetre lesions, but did not investigate shorter acquisition durations than 2 min. In our study, we observed that shorter acquisition times warrant slightly higher β-factors. In fact, we saw that a β-factor of 200 was a valid option for 1–5 min acquisitions but would result in a significant overestimation of RC for shorter acquisition times.

Quantitation of dynamic PET data involves noise reduction in the curve fitting process included in the pharmacokinetic analysis, making high CV_RC_ less problematic than in static exams. Nevertheless, CV_RC_ values are high at low-count statistics, in particular in small structures (Fig. 3). The smoothing effect of curve fitting will also be less helpful in areas of the TACs with rapid changes, and in particular near maxima because a sharp peak may only span one frame. This is particularly relevant in the special case of brain imaging with an IDIF derived from the intracranial ICA. The IDIF increases immediately after the bolus injection and peaks during the short frames at the start. When comparing count statistics in the clinical data with the phantom data, we see that the count statistics in the peak of the blood pool TAC, with 10-s frames, matches the count statistics in the 30-s frame in the phantom. We also see that the count statistics in the tumour VOI matches the count statistics in the 5-min frames in the phantom. The β-factor could be optimized individually for different frames lengths, leading to several β-factors in a dynamic dataset. This will most likely not be applied in clinical routine, but could be applied in a research protocol.

The AUC of the input function reflects the total concentration of tracer in blood and is a key component in the pharmacokinetic analysis of dynamic PET data. In our clinical data, we found that increasing the β-factor reduced the AUC of the intracranial ICA-segmented input function, primarily due to increased PVE. However, these effects were less pronounced than those observed in the 5-mm sphere, where RC changed more than 70% in this β range for the 10-s frames, and about 39% in the 5-min frames. While PVE correction could provide a more robust IDIF, we opted not to apply it in this study to focus on the direct impact of β-factors. Different tracers lead to varying target region dynamics, but typically the TAC increases slower than for the blood pool TAC before either dropping back down or going into a plateau. With respect to frame durations, the IDIF and tumour provide AUC at different ends of the protocol and will be affected differently by the β-factor. Nevertheless, our clinical data showed similar β-factor dependency for both the IDIF and lesions, suggesting that some dependencies on count statistics may offset each other in practice. This β-factor effect on Ki, while moderately strong, should be considered when designing protocols and interpreting findings, as a 20% change may or may not be clinically significant. Ki values offer diagnostic and staging insights, along with tracking disease progression and treatment response.

Several study limitations should be noted. Firstly, in clinical dynamic PET scans, tracer concentration varies in tissues and blood, unlike in the constant conditions of the phantom study. Previous studies have shown that tissue to background ratio affects the choice of β-factor [10, 18]. Secondly, the phantom study is based on spheres of specific sizes, which may not directly apply to voxel-based quantification but still informs analysis of structures of variable size. Thirdly, the phantom’s small size (20 cm diameter) may not represent whole-body scans, especially in larger patients with potentially higher noise levels [19]. Fourthly, an absolute quantification of tracer kinetics requires a metabolite-corrected plasma function as an input function. Even though the IDIF extracted in the clinical example represents radioactivity concentration in whole blood, it will not affect the scope of this study because the same method was applied to all β-factors. Lastly, we used a 10% peak RC measurement, which should be considered when interpreting results. Despite these limitations, our findings can guide optimized reconstruction protocols for dynamic PET quantification.

## Conclusion

This study showed that the choice of β-factor impacts the quantitation of dynamic PET scans. A favourable β-factor provides a balance between accuracy and variability. However, the accuracy will be the most prominent metric in a pharmacokinetic analysis of dynamic PET data. Based on these results, we suggest a β-factor of 300–500 for quantitation of small structures with dynamic PET imaging, while larger structures may benefit from higher β-factors.

## Data Availability

The phantom data and MATLAB code used during the current study are available from the corresponding author on reasonable request. The clinical data included in the present study are part of an ongoing clinical trial at Oslo University Hospital and will be shared at study end.

## Abbreviations

AUC: Area under curve
CR: Contrast recovery
CT: Computed tomography
CV: Coefficient of variation
FOV: Field of view
ICA: Internal carotid artery
IDIF: Image derived input function
NEMA: National electrical manufacturers association
OSEM: Ordered subset expectation maximation
PET: Positron emission tomography
PSF: Point spread function
PSMA: Prostate-specific membrane antigen
PVE: Partial volume effect
RC: Recovery coefficient
RMSE: Root-mean-square error
SD: Standard deviation
SUV: Standardized uptake value
TAC: Time activity curve
TOF: Time of flight
VOI: Volume of interest
